# The effect of impulsivity and inhibitory control deficits in the saccadic behavior of premanifest Huntington’s disease individuals

**DOI:** 10.1186/s13023-019-1218-y

**Published:** 2019-11-08

**Authors:** Filipa Júlio, Gina Caetano, Cristina Januário, Miguel Castelo-Branco

**Affiliations:** 1 CIBIT - Coimbra Institute for Biomedical Imaging and Translational Research, University of Coimbra, Coimbra, Portugal; 2 Faculty of Psychology and Education Sciences, University of Coimbra, Coimbra, Portugal; 3 CNC.IBILI ― Center for Neuroscience and Cell Biology/Institute for Biomedical Imaging and Life Sciences, Faculty of Medicine, University of Coimbra, Coimbra, Portugal; 4 IBEB – Institute of Biophysics and Biomedical Engineering, Faculty of Sciences, University of Lisbon, Campo Grande, 1749-016 Lisbon, Portugal; 5 CHUC – Coimbra University Hospital, Coimbra, Portugal; 6 ICNAS - Institute of Nuclear Sciences Applied to Health, University of Coimbra, Coimbra, Portugal

**Keywords:** Huntington’s disease, Cognition, Oculomotor function, Inhibitory control, Impulsivity

## Abstract

**Background:**

This study aims to test response inhibition in premanifest Huntington’s disease individuals (Pre-HD), in the context of a saccadic paradigm with working memory demands and fronto-executive load as a way to measure inhibitory control deficits and impulsive behavior in Huntington’s disease (HD).

**Methods:**

The oculomotor function of 15 Pre-HD and 22 Control individuals was assessed using an experimental paradigm comprising four horizontal saccadic tasks: prosaccade (PS), antisaccade (AS), 1- or 2-back memory prosaccade (MPS), and 1- or 2-back memory antisaccade (MAS). Success rate, latency, directional and timing errors were calculated for each task. A comprehensive battery of neuropsychological tests was also used to assess the overall cognitive functioning of study participants. Statistical correlations between oculomotor, clinical and cognitive measures were computed for the Pre-HD group.

**Results:**

Pre-HD participants showed reduced success rate in the AS task, increased direction errors in the AS and MAS tasks and decreased latency in the MAS task when compared to Controls, despite presenting similar executive and memory scores in the conventional neuropsychological tests applied. Significant associations were identified between specific AS and MAS parameters and disease-related measures, cognitive skills and other oculomotor results of Pre-HD participants.

**Conclusions:**

Our results show that oculomotor performance in premanifest Huntington’s disease deteriorates once inhibitory control, working memory and/or fronto-executive load are added to the task. A more automatic pattern of performance, including a faster response time and directionally erroneous eye movements were detected in the oculomotor behavior of the Pre-HD group—these alterations were significantly correlated with disease stage and cognitive status. Our saccadic paradigm was able to capture impulsivity and inhibitory control deficits in a group of Pre-HD individuals on average far from symptom onset, thus holding the potential to identify the earliest disease-related changes.

## Background

Huntington’s disease (HD) is an autosomal inherited neurodegenerative disorder caused by a Cytosine-Adenine-Guanine (CAG) trinucleotide repeat expansion in the huntingtin gene. HD is characterized by motor abnormalities, emotional and behavioral changes, and a general cognitive decline [1–3]. Testing positive for HD supplies only information on gene status, but not on disease state, as the test result gives little indication on how and when the triad of symptoms will start [4, 5]. The proper identification and quantification of the signs and symptoms exhibited by individuals that tested positive for HD but are still in a premanifest stage is vital to implement and assess the efficacy of any therapeutic interventions [6].

Although there is now a consensual idea that cognitive impairments emerge years before HD clinical diagnosis and that the progression of cognitive decline is gradual [7–10], the conversion of an individual from a premanifest to a manifest HD status is classically defined solely on the basis of motor signs, with no consideration for cognitive and/or psychiatric disturbances [1, 2, 11]. Nevertheless, the cognitive changes associated with HD need also to be fully addressed in disease progression and characterization [12]—impairments in inhibitory control, attention, working memory, executive functions, mental flexibility, psychomotor functions, planning, processing speed, multitasking, organization, problem solving, implicit learning, visuospatial functions, timing and movement sequencing, face and emotion processing and recognition [4, 9–11, 13–25].

One of the cognitive symptoms most peculiar to HD is the executive dysfunction syndrome, a condition that encompasses disinhibition, attentional deficits, poor impulse control, and perseveration [12, 26]. In HD, these changes in different aspects of top-down control mechanisms are associated with the disruption of the corticostriatal circuitry, especially the prefrontal-striatal connections [19, 27–30]. This circuitry is important for the planning of an instrumental performance, temporal control over motor output, and response inhibition in general [27, 28, 31]. Accordingly, individuals with established basal ganglia damage, such as those with HD or Parkinson’s Disease (PD), experience difficulty selecting a preferred motor activity and inhibiting undesired responses, frequently displaying impulsivity and altered behavior inhibition in their performance [29, 32–37].

Thus, impulsivity can be defined as the observable behavioral manifestation of a failure of the prefrontal cortex in inhibiting an overt motor act or response [28]. Despite the multitude of studies about impulsivity in PD [36–39], impulsive behavior in HD needs to be further investigated. Harrington et al. [8] pinpoint that fact, referring to a large, multi-centered prospective study (PREDICT-HD) to indicate that one of the domains that has been inadequately assessed in HD is inhibition. Also, as stated by Bari and co-authors [28], there are many unanswered questions about the mechanisms underlying abnormal impulsive behavior.

Considering these outstanding questions, we aimed at assessing response inhibition and, hence, impulsivity, in an objective manner, by using an oculomotor paradigm with a component of inhibitory motor control and increasing cognitive load. Oculomotor impairments are precisely among the first manifestations of HD, with saccadic abnormalities having been frequently described in HD patients [1, 4, 22, 40–47]. Although mixed findings have been reported about premanifest HD individuals’ oculomotor performance [4, 40–43, 48–50], studies have shown significant alterations in antisaccade and memory-guided saccade measures of latency, higher variability of saccade latency and increased error rates [4, 40, 43, 45, 50]. Findings include higher disinhibition (impaired saccade suppression), higher number of anticipatory saccades (that is, timing errors), increased errors in memory-guided saccade tasks, prolonged latency for initiating voluntary saccades, and an increase in latency for reflexive prosaccades [40, 41, 46, 48, 51]. Nevertheless, Gorges et al. [33] suggest that a comprehensive explanation for the lack of inhibition control at the saccadic or eye movement level in HD remains to be identified. Saccadic paradigms designed to assess inhibition and impulsivity processes in HD can further help to identify underlying deficits and mechanisms. Also, most cognitive/executive tasks, including those explicitly devised as a measure of behavioral inhibition, have been criticized for suffering from low reliability [28]. Thus, as stated by Zhang and colleagues [37], the use of saccadic measures to test deficits in inhibitory oculomotor control with an emphasis on impulsive response patterns can benefit the objective assessment of this cognitive and behavioral trait.

Finally, a number of studies suggest that task complexity (higher cognitive/executive load) is essential for discriminating Pre-HD individuals and controls in the majority of saccadic paradigms [44, 46, 51]. The known frontostriatal impairment in Huntington’s disease, and the proven influence of this circuitry in the inhibitory component of antisaccades, imply that increasingly complex executive and memory saccadic tasks are expected to be more sensitive to disease onset than simple ones [52–54].

This study aims to test if inhibitory control demanding oculomotor paradigms, embedded with an increasing fronto-executive and memory load, may provide a sensitive and objective measure of impulsivity, hence failure in inhibiting a motor act, in premanifest HD individuals.

## Methods

### Participants

Thirty-seven participants completed the neuropsychological assessment and thirty-six participants completed the saccade/eye-tracking protocol (due to technical problems, the oculomotor data of one Pre-HD participant could not be recorded).

Study participants were primarily recruited from the Neurological Department – Movement Disorder Unit of Coimbra University Hospital. They were also recruited through the Huntington’s disease Portuguese Association. All participants gave their informed written consent after the study protocol had been explained to them. Informed consent was obtained according to the Declaration of Helsinki and all procedures were approved by the local Ethics Committee (Faculty of Medicine, University of Coimbra).

Exclusion criteria included history of alcohol or drug abuse/dependence, concurrent neurological illness, severe ophthalmic disease, and use of psychotropic medication (the last criterion only applied to Controls). The Montreal Cognitive Assessment test, a mild cognitive impairment and dementia screening tool, was also an exclusion criterion [55, 56]—a below the established normative reference score based on age and education [57] was presumed to indicate the presence of mild cognitive impairment and, thus, the participant would no longer take part in the study.

Clinical history, current medications (see Additional file 1: Table S1), and any other information considered to be important for taking part in this study were registered as well. Participants were assigned to two groups (see Table 1):

**Table 1:** Demographic characteristics of the CTRL and Pre-HD groups

	CTRL = 22		Pre-HD = 15		Chi-Square /Mann-Whitney	
					*χ*^*2*^*/ U*	*p*
	Gender (F:M) 15:7		Gender (F:M) 8:7		0.836	0.361
	Handedness (R:L) 19:3		Handedness (R:L) 15:0		3.058	0.383
	Median	IQR	Median	IQR		
Age (years)	34	12	37	12	161.5	0.914
Education (years)	11.5	2	12	7	155.5	0.766
CAG repeats	–	–	41	2	–	–
Time to HD Onset (years)	–	–	21.1	11	–	–
UHDRS - TMS	–	–	1	3	–	–
UHDRS - OculoTMS	–	–	0	1	–	–
UHDRS-TFC	–	–	13	0	–	–

No significant differences were found between Pre-HD and Controls in any of the Demographic variables

*IQR* Interquartile Range, *CAG repeats* CAG repeat expansion confirmed by a genetic test, *Time to HD Onset* estimated number of years to the formal diagnosis of manifest HD, calculated with the Langbehn’s formula [58], *UHDRS* Unified Huntington’s Disease Rating Scale [59], *TMS* Total Motor Score of the UHDRS, *OculoTMS* a composite score extracted from the sum of the oculomotor items of UHDRS-Motor scale, *TFC* Total Functional Capacity scale of the UHDRS

Premanifest gene carriers (Pre-HD): 15 individuals with an expanded HD gene (≥36 CAG repeats) who demonstrated either no signs or soft signs of motor abnormalities, i.e., had a diagnostic confidence score of 0–3 on the Unified Huntington’s Disease Rating Scale – Motor scale (UHDRS-Motor), a Total Motor Score (TMS) of ≤5, and a Total Functional Capacity (TFC) score of 13 in this UHDRS subscale [59].

Controls (CTRL): 22 non-gene carriers, defined as those individuals with two unexpanded HD alleles (< 36 CAG repeats - gene negative status), or healthy volunteers who were not at risk for HD and had no known neurological disorder (spouses and healthy participants from the community).

### Clinical evaluation

An experienced movement disorder neurologist administered the motor subscale of the Unified Huntington’s Disease Rating Scale [59] to the Pre-HD participants to establish, with at least 99% certainty, whether individuals had motor manifestations of HD. The neurologist assigned an overall confidence rating that represented the likelihood of motor abnormalities be attributable to HD. The individuals with a Total Motor Score (TMS) of ≤5 and a rating from 0 to 3 in the diagnostic confidence score were classified as Pre-HD. A higher TMS indicates worse clinical symptoms. A cut-off of 5 points was used to determine the premanifest status of the participant, in accordance with the EHDN – Registry study’s guidelines [60]. A composite score (OculoTMS) was computed from the oculomotor component of the UHDRS-Motor scale—ocular pursuit, saccade initiation and saccade velocity items. The Total Functional Capacity subscale (TFC) of the UHDRS was also administered to all the participants of the clinical group, to assess their functional status and determine their premanifest HD stage [45, 59]. The TFC uses a rating between 0 and 13 of different functional domains, and a higher score means higher autonomy and independence in the activities of daily living.

### Oculomotor experiment

**Fig. 1:**
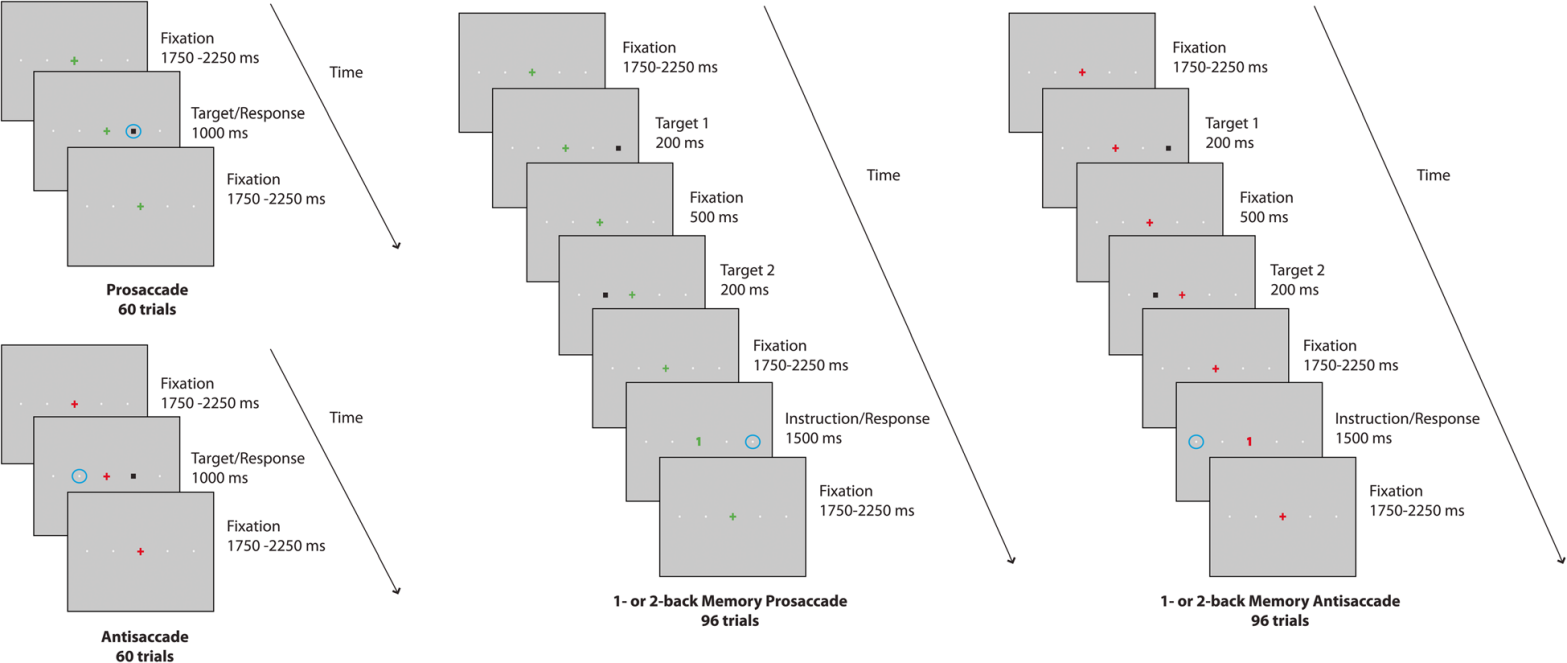
Experimental design of the four horizontal saccadic tasks

Participants had to complete four horizontal saccadic tasks, where saccadic movements were recorded using an iViewX Hi-speed eye tracking system (1.06, Sensor Motoric Instruments, Teltow) – see Fig. 1. This paradigm was designed taking into account former findings in healthy individuals, that showed specific disruption of saccadic inhibition when the oculomotor task was conjoint to an increasing executive load via an n-back memory task [52, 54].

#### Oculomotor testing procedure

Participants were seated in front of a 17-in. monitor with their heads placed in a stable chin rest that was positioned 52 cm from the screen. Prior to each oculomotor task, the examiner instructed verbally the participant to ensure that the goal of each task was fully understood, followed by a practice block to discard potential novelty effects in task performance. Eye-tracking recordings were performed after a 9-point calibration using the subject’s dominant eye. The saccade protocol was administered over a period of 40 to 60 min, in a fixed order, with an increasing working memory and fronto-executive load.

The experiment was compound of four conditions. In each, a central fixation point was defined (cross, 1^o^ diameter in visual angle), and peripheral visual targets (black square, 0.6^o^ visual angle) were randomly assigned to four possible positions at ±6^o^ or ± 12^o^ visual angle. Small position cues were present throughout the experiment at each of the four possible target positions (* symbols, 0.24 ^o^ visual angle, light gray in color).

Prosaccade task (PS): The participant was instructed to fixate gaze on a central illuminated green cross, and to look to the peripheral target as rapidly as possible once it appeared, and then return to the central fixation cross. This task consisted of 60 trials.

Antisaccade task (AS): The participant was instructed to fixate gaze on a central illuminated red cross, and to look to the opposite direction of the visual target once it appeared, i.e., the mirror-image location of the target at an equal distance from the central fixation cross. Afterwards, the participant was asked to return to the central fixation cross. This task also consisted of 60 trials.

1- or 2-back Memory Prosaccade task (MPS): The participant was instructed to fixate gaze on a central green cross. While two peripheral squares appeared, the participant was asked to continue to fixate the central green cross. The task period was assigned once the central fixation cross was replaced by a digit, either a green one or a green two, when the participant had to generate a saccade to a remembered position. If the digit was one, the participant was asked to look at the remembered position where the first square had appeared. If the digit was two, the participant was asked to look at the remembered position where the second square had appeared. Then, the participant had to return to the central fixation cross. This task consisted of 96 trials.

1- or 2-back Memory Antisaccade task (MAS): The participant was instructed to fixate gaze on a central red cross. The task period was assigned once the central fixation cross was replaced by a digit, either a red one or a red two, when the participant had to generate a saccade. If the digit was one, the participant was asked to look to the opposite direction (i.e., the mirror-image location) of the remembered position where the first square appeared. If the digit was two, the participant was asked to look to the opposite direction of the remembered position where the second square appeared. Then, the participant had to return to the central fixation red cross. This task also consisted of 96 trials.

#### Oculomotor data processing

Regarding the psychophysics task, BeGaze software (3.4, Sensor Motoric Instruments, Teltow) was used to create experiments based on saccade detection: peak velocity threshold 40^0^/ms; saccade velocity initiation and termination of 15^0^/ms and 85^0^/ms, respectively; minimum fixation duration of 50 ms; minimum saccade duration of 22 ms. Computed data on saccades and blinks were extracted and further analyzed with the Matlab software toolbox (R2013a).

Identification of valid trials for each task was performed applying the following criteria: *i)* initiation and termination had to be within a region of interest (± 2.5^0^ x ± 4^0^ visual angle) of the fixation and target position, respectively; *ii)* the primary saccade initiated within the central fixation ROI, had an amplitude enabling termination outside the ROI (horizontally), was performed in the correct direction, and had a latency higher than 80 ms; *iii)* if the latency was below 80 ms it was considered an anticipatory saccade error (latency-type error); *iv)* if the saccade was performed in the opposite horizontal direction it was considered a direction error; *v)* the primary saccade had a latency below 700 ms (PS and AS tasks) or 1000 ms (MPS and MAS tasks), otherwise it was considered a long-latency error (latency-type error); *vi)* the total saccadic movement finished within the ROI for the intended target position, prior to return to the central fixation position. Additionally, trials contaminated by blinks or other abnormalities were discarded from the analysis.

For every participant, measures were computed for each of the PS, AS, MPS and MAS tasks, namely: percentage of successful trials – trials free of errors; percentage of direction errors – resulting from a reflexive saccade in the opposite direction of the correct hit; percentage of anticipatory saccade errors – resulting from a premature saccade, in which the participant took less than 80 ms to start the saccade; latency – saccadic reaction time, that is time from stimulus appearance to the onset of the primary saccade (milliseconds). The calculation of mean latencies included only correct trials that met the inclusion criteria.

Only participants that had at least 25% of successful trials (i.e., trials free of any kind of error type) were included in the analysis, for each of the oculomotor tasks (see Additional file 2: Table S2).

### Neuropsychological assessment

We have used a comprehensive neuropsychological test battery that was designed to maximize sensitivity to the frontostriatal neural circuitry and cognitive control abilities, and that mainly incorporated widely used executive and memory tests (see Table 2). We aimed at tapping the major cognitive functions known to be affected in the early stages of HD [10, 11, 18, 22, 60, 77]. We computed two main composite scores from this battery—Executive and Memory—to have a baseline depiction of the cognitive abilities involved in the saccadic paradigm created for this study, and to acknowledge any significant differences between the overt cognitive profile of Pre-HD and Control participants that could otherwise explain the potential differences found in their oculomotor behavior.

**Table 2:** Neuropsychological assessment protocol

Tool	Goal/Assessment domain
Montreal Cognitive Assessment - MoCA [55, 56]	mild cognitive impairment and dementia screening
Stroop Test - Color Naming, Word Reading, and Interference tasks [61, 62]	executive function – cognitive flexibility and processing speed
Edinburgh Handedness Inventory - Portuguese adaptation [63]	handedness definition
Digit Symbol subtest of the Wechsler Adult Intelligence Scale-III [64, 65]	psychomotor speed and working memory
Rey Auditory Verbal Learning test - Portuguese version [66, 67] - total trials 1–5	verbal memory
12-item short form of the Raven Advanced Progressive Matrices [68]	indication of non-verbal intelligence and to control for individual differences in IQ that are unrelated to illness
Corsi Block-Tapping task [69, 70]	psychomotor speed, working memory and executive functioning - the product of the total number of correct trials and the length of the largest sequence was calculated
Benton Visual Retention test [71]	visual memory
Rey Auditory Verbal Learning test - Portuguese version [66, 67]– recall and recognition trials	verbal memory
Benton Visual Form Discrimination test [72]	visual perception
Phonemic Verbal Fluency test: 3 letters – P, M, R [73]	executive function – working memory, word generation and inhibition
Semantic Verbal Fluency test: category – animals [74]	executive function – working memory, word generation and inhibition
Vocabulary of the WAIS-III [64, 65]	indication of verbal intelligence and control for individual differences in intelligence that are unrelated to illness
Hospital Anxiety and Depression Scale – Snaith Irritability Scale (HADS-SIS) [75, 76]	psychiatric symptoms and prevalence of depression and anxiety

An Executive Composite Score was computed from six neuropsychological test scores [Stroop word reading test – total correct; Stroop color naming test – total correct; Stroop interference test – total correct; Symbol digit modality test – total correct; Verbal fluency test (letters-PMR) – total correct; Verbal fluency test (category-animals) – total correct]. A Memory Composite Score was computed from six neuropsychological test scores [Benton visual retention test – total correct; Auditory verbal learning test (trials-1-5) – total correct; Auditory verbal learning test (recall) – total correct; Auditory verbal learning test (recognition) – total correct; Corsi block tapping task (direct) – total correct; Corsi block tapping task (inverse) – total correct].

Additionally, we have assessed the global cognitive status, the verbal and non-verbal intelligence level, the visual perception abilities, and the neuropsychiatric symptoms of study participants using standardized measures of these domains.

The neuropsychological battery was administered over a period of one and a half hours, in a strictly prescribed order, to avoid interference problems related to evaluating the same contents or assessing the same domain in several tasks in a row, and to respect the time intervals required by certain tests.

### Statistics

Statistical analysis was performed with the software IBM SPSS Statistics, version 24, adopting a level of significance of α = 0.05, and only significant results were reported and further debated in the “Results” and “Discussion” sections.

Outliers were excluded from data analysis for each oculomotor parameter in the four saccadic tasks―values below Q1–1.5xIQR and above Q3 + 1.5xIQR (see Additional file 3: Table S3). When comparing the neuropsychological and saccadic performance of Pre-HD and Control groups, ANCOVA statistical analysis was performed with age as a covariate, given that this variable is known to affect cognition and reflexive and voluntary eye movements both in clinical and healthy populations [43, 48, 78, 79]. Mann-Whitney U tests were used to compare the demographic variables of the two groups. Comparisons of nominal/categorical variables between groups were performed resorting to Chi-square tests of independence. Wilcoxon-Signed rank tests were used to further examine the effects of task condition (PS, AS, MPS and MAS) in the Pre-HD participants’ saccadic performance. Spearman rank correlation coefficients were calculated to analyze the associations between the performance of the Pre-HD participants in the oculomotor measures where a group difference was found and other clinical, cognitive and oculomotor data of the Pre-HD group. Benjamini–Hochberg corrections with false positive rate established at 0.05 were used to deal with multiple comparisons.

## Results

The Pre-HD and CTRL participants enrolled in our study were matched in terms of age, education level, gender, and handedness **(**see Table 1**).**

### Oculomotor results

**Fig. 2:**
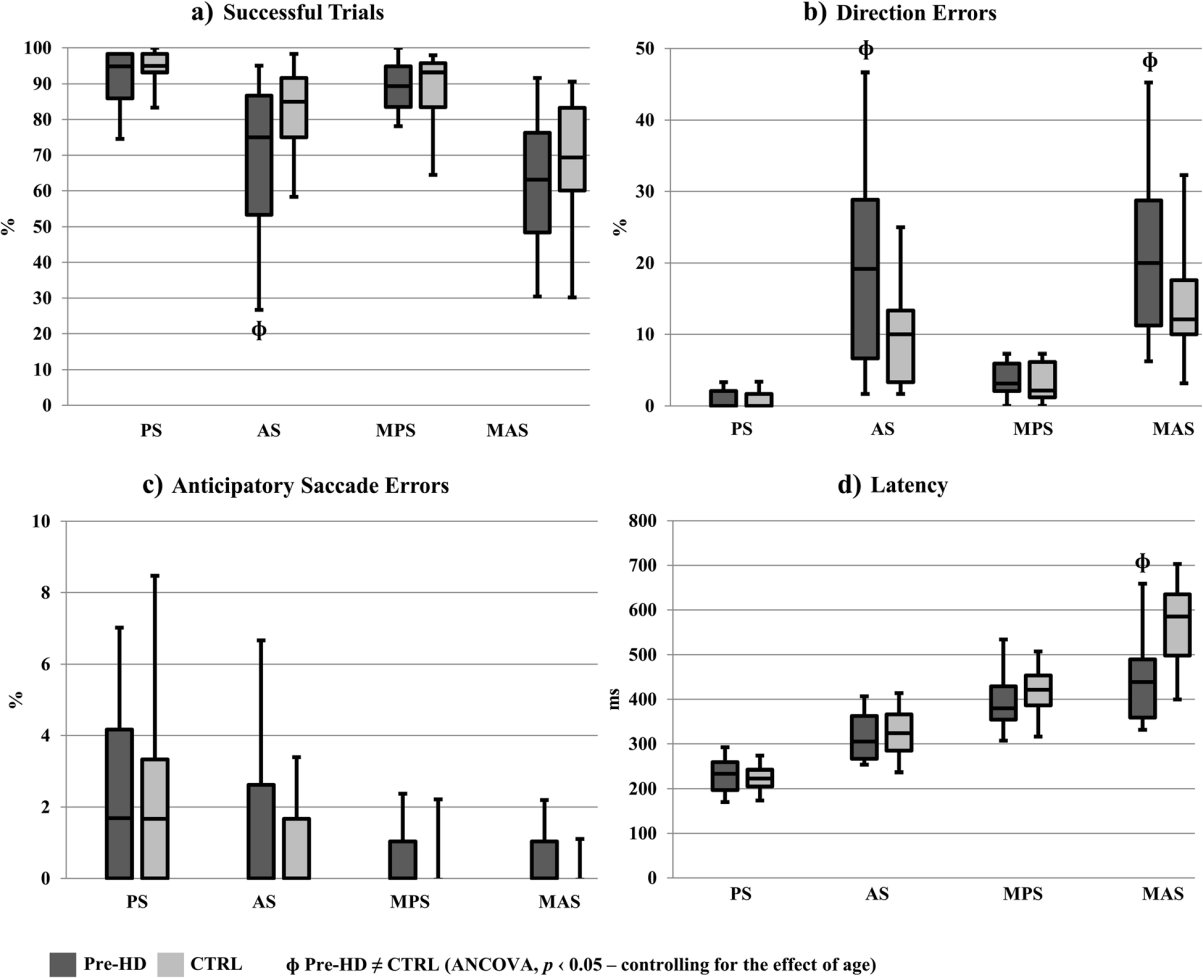
Oculomotor results of the CTRL and Pre-HD groups across the four saccadic tasks. Box plot (line, median; box, 1st and 3rd quartiles; whiskers, minimum and maximum). PS – Prosaccade; AS – Antisaccade; MPS – 1- or 2-back memory Prosaccade; MAS – 1- or 2-back memory Antisaccade. **a** Successful trials – trials free of errors (%); **b** Direction errors – resulting from a reflexive saccade in the opposite direction of the correct hit (%); **c** Anticipatory saccade errors – resulting from a premature saccade: participant takes less than 80 ms to start the saccade (%); **d** Latency – saccadic reaction time: time from stimulus appearance to the onset of the first saccade (milliseconds). **ɸ** Pre-HD ≠ CTRL (ANCOVA, p ‹ 0.05 – controlling for the effect of age)

The comparison of the saccadic performance of the two groups (see Fig. 2 and Table 3) revealed that alterations of oculomotor performance were present in the clinical group compared to controls, especially in the tasks with higher executive and/or memory load, namely the AS and MAS tasks. In addition, the analysis of the performance of Pre-HD participants across the four saccadic conditions revealed that both accuracy and timing measures reflected the impact of the incremental executive and memory demands of the saccadic tasks (see Additional file 4: Table S4).

**Table 3:** Comparison of the Oculomotor results of the CTRL and Pre-HD groups across the four saccadic tasks

	% Successful trials		% Direction errors		% Anticipatory saccade errors		Latency	
	*F*	*p*	*F*	*p*	*F*	*p*	*F*	*p*
PS	(1,30) 3.299	0.079	(1,31) 0.203	0.655	(1,31) 0.124	0.728	(1,32) 0.954	0.336
AS	(1,32) 5.200	0.029*	(1,31) 7.278	0.011*	(1,31) 0.984	0.329	(1,32) 0.132	0.719
MPS	(1,30) 0.081	0.778	(1,30) 0.158	0.694	(1,30) 0.516	0.478	(1,31) 1.186	0.285
MAS	(1,30) 1.287	0.266	(1,28) 5.480	0.027*	(1,27) 1.984	0.170	(1,29) 12.272	0.002*

*PS* Prosaccade, *AS* Antisaccade, *MPS* 1- or 2-back memory Prosaccade, *MAS* 1- or 2-back memory Antisaccade

*Pre-HD ≠ CTRL (ANCOVA, *p* ≤ 0.05 – controlling for the effect of age)

For the percentage of successful trials, an important statistically significant difference was found between Pre-HD and CTRL participants in the AS condition (*F*(1,32) = 5.200, *p* = 0.029). This result suggests that once an executive load or inhibitory demand was introduced into an otherwise simple reflexive saccadic task, the Pre-HD group started to show an abnormal oculomotor behavior, with a significant decrease in their success rate due to the switch in the protocol.

Notably, for the percentage of direction errors, again a statistically significant difference was found between Pre-HD and CTRL participants in the AS condition (*F*(1,31) = 7.278, *p* = 0.011) and in the MAS condition (*F*(1,28) = 5.480, *p* = 0.027). These results suggest that, when an executive load is added to the task, either independently or combined with memory demands, the oculomotor performance of Pre-HD participants fails to adapt to the new goal and inhibition deficits emerge.

For the percentage of anticipatory saccade errors, no statistically significant differences were found between Pre-HD and CTRL participants across the four saccadic conditions. These results suggest that both groups exhibit a similar rate of premature saccades along the different task conditions, albeit the reduced accuracy displayed by Pre-HD participants in the more demanding AS and MAS tasks.

Finally, for the primary saccade latency, a statistically significant difference was found between Pre-HD and CTRL participants in the MAS condition, where Pre-HD participants showed a faster saccadic reaction time compared to controls (*F*(1,29) = 12.272, *p* = 0.002). These findings suggest that for the premanifest HD participants, latency in the context of the most demanding saccadic condition can illustrate a more automatic response pattern when the task demands increase.

The analysis of the saccadic performance of the Pre-HD participants across the four different task conditions (PS, AS, MPS and MAS) revealed significant effects of the increasing cognitive load in the percentage of successful trials, percentage of direction errors, percentage of anticipatory errors and latency (see Additional file 4: Table S4). Latency and the percentage of directions errors seemed to be particularly sensitive measures for capturing the decremental impact of the increasing executive and memory demands of the oculomotor task on the behavior of premanifest HD participants [all significant differences |*Z|* ≥ 2.803, *p* ≤ 0.05]. Interestingly, only the primary saccade latency differed between the AS and MAS oculomotor performance of the Pre-HD group (*Z* = − 3.059, *p* = 0.002), which suggests that in saccadic conditions with inhibition demands the behavior of the clinical group is globally similar (and equally compromised), whereas a more automatic response pattern emerges when the task demands increase (MAS task).

### Neuropsychological results

No significant differences were found between Pre-HD and CTRL participants in the Executive and Memory Composite scores computed from the neuropsychological battery, nor in any of the other neuropsychological and neuropsychiatric measures used (see Table 4). These results suggest that both groups had a similar cognitive and psychiatric status, as assessed with conventional tests and scales, which might indicate that the oculomotor differences found between the two groups cannot be explained by disparate executive, memory or psychiatric conditions.

**Table 4:** Neuropsychological test results of the CTRL and Pre-HD groups

	CTRL		Pre-HD		ANCOVA	
	Median	IQR	Median	IQR	*F*	*p*
Executive Composite Score	321	65	307	74	(1,34) 0.313	0.579
Memory Composite Score	206.5	32	203	61	(1,34) 0.225	0.638
Vocabulary - WAIS III	36.5	15	37	13	(1,34) 0.271	0.606
Raven Matrices	8	2	9	3	(1,34) 0.105	0.748
Montreal Cognitive Assessment	26	3	26	3	(1,34) 0.248	0.622
Benton Visual Form Discrimination	30.5	3	30	4	(1,34) 0.310	0.861
HADS-SIS – Depression	4	5	4	6	(1,34) 0.150	0.700
HADS-SIS – Anxiety	6	7	5	5	(1,34) 0.197	0.660

No significant differences found between Pre-HD and Controls in any of the Neuropsychological Measures

*IQR* Interquartile Range, *HADS-SIS* Hospital Anxiety and Depression Scale – Snaith Irritability Scale

Executive Composite Score = Stroop word reading test (total correct) + Stroop color naming test (total correct) + Stroop interference test (total correct) + Symbol digit modality test (total correct) + Verbal fluency test (letters-PMR) (total correct) + Verbal fluency test (category-animals) (total correct)

Memory Composite Score = Benton visual retention test (total correct) + Auditory verbal learning test (trials-1-5) (total correct) + Auditory verbal learning test (recall) (total correct) + Auditory verbal learning test (recognition) (total correct) + Corsi block tapping task (direct) (total correct) + Corsi block tapping task (inverse) (total correct)

### Correlational analysis

In the Pre-HD group, the four oculomotor parameters that statistically differed from the CTRL group were significantly correlated with their results in other clinical, cognitive and oculomotor measures included in our study protocol (see Table 5 and Additional file 5: Table S5). Importantly, the percentage of direction errors of the Pre-HD group in the AS and MAS conditions were significantly correlated with the UHDRS-OculoTMS (*r*_*s*_ = 0.533, *p* = 0.049 and *r*_*s*_ = 0.609, *p* = 0.027, respectively), which reflects oculomotor abnormalities detected at neurological examination. Moreover, the Pre-HD primary saccade latency in the MAS condition was significantly correlated with the Time to HD Onset (*r*_*s*_ = − 0.620, *p* = 0.032). Finally, we have found that the memory composite score was significantly associated with the AS percentage of successful trials and the MAS percentage of direction errors in the Pre-HD group (*r*_*s*_ = 0.533, *p* = 0.050 and *r*_*s*_ = − 0.660, *p* = 0.014, respectively). These results suggest that changes in specific oculomotor parameters prior to the onset of clinically relevant motor disturbances are significantly associated with important disease-related features and cognitive skills in premanifest HD individuals. Additionally, the significant associations found between antisaccade trajectory and timing measures in the Pre-HD group indicate that executively demanding oculomotor tasks seem to induce a consistently erroneous and impulsive saccadic behavior in premanifest HD individuals.

**Table 5:** Correlations between the oculomotor, clinical and cognitive results of the Pre-HD group

Pre-HD	AS Successful trials *n* = 14		AS Direction errors *n* = 14		MAS Direction errors *n* = 13		MAS Latency *n* = 12	
	*rho*	*p*	*rho*	*p*	*rho*	*p*	*rho*	*p*
CAG	−0.089	0.763	0.000	1.000	−0.220	0.469	0.303	0.339
Time to HD Onset (years)	0.420	0.135	−0.310	0.281	−0.014	0.964	−0.620	0.032*
UHDRS-TMS	−0.356	0.212	0.314	0.274	0.284	0.347	−0.096	0.766
UHDRS-OculoTMS	−0.473	0.088	0.533	0.049*	0.609	0.027*	0.096	0.767
Executive Score	0.459	0.099	−0.257	0.375	−0.325	0.279	0.133	0.681
Memory Score	0.533	0.050*	−0.300	0.298	−0.660	0.014*	0.186	0.564

*AS* Antisaccade, *MAS* 1- or 2-back memory Antisaccade

*CAG repeats* CAG repeat expansion confirmed by a genetic test; Time to HD Onset – estimated number of years to the formal diagnosis of manifest HD, *UHDRS* Unified Huntington’s Disease Rating Scale, *TMS* Total Motor Score of the UHDRS, *OculoTMS* a composite score extracted from the sum of the oculomotor items of UHDRS-Motor scale

*Correlation is significant at 0.05 level (two-tailed)

## Discussion

The current study addressed the role of saccadic movement parameters, and specifically saccadic inhibition with or without memory and fronto-executive load, as a potential marker of impulsive behavior in premanifest Huntington’s disease. We hypothesized that an oculomotor experiment embedded with a cognitively demanding paradigm [28, 52, 54], aimed at increasing fronto-executive load whilst tapping onto the inhibitory component of saccadic eye movements, could be more sensitive in detecting the earliest HD-related alterations than formerly investigated paradigms [42, 46, 49, 51, 80, 81], conventional cognitive tests that evaluate executive function and working memory [61, 62, 69, 70, 73, 74], and standard clinical evaluation of oculomotor function [59, 81]. Particularly, the influence of frontal-executive load in oculomotor inhibition processes was analyzed in this study and tested as a potential trigger of impulsive response patterns in premanifest HD individuals.

We have found that Pre-HD participants with a similar executive and memory performance in conventional tests to controls, show statistically significant saccadic impairments in an oculomotor paradigm that encloses inhibition and increasing cognitive demands.

The Pre-HD group has shown impairments particularly in oculomotor tasks with an inhibitory component, exhibiting a decreased success rate in the AS task, a higher percentage of direction errors in the AS and MAS tasks, and a reduced response latency in the MAS condition when compared to controls. The timing and trajectory abnormalities shown by the Pre-HD group of our study illustrate the impaired saccade suppression in premanifest HD reported by Anderson & MacAskill [40], the higher incidence of unusually early saccades in premanifest individuals reported by Antoniades et al. [48, 51], and the HD patients’ inability to stifle saccades especially in highly demanding memory and executive tasks reported by Ali et al. [82]. The lower percentage of success rate in the AS task and the reduced saccade latency shown by the premanifest HD group in the MAS task may be interpreted as indicators of more impulsive oculomotor behavior / automatic response pattern due to early impairments in inhibitory control mechanisms. Farrow et al. [83] suggested that in cognitive tasks with increasing executive load, premanifest HD individuals have greater difficulty overcoming the more demanding executive conditions and are more likely to inappropriately make more automatic responses. Our data seems to be in line with this statement—control participants seem to show a stable oculomotor performance along the four different saccadic tasks, increasing their response latency in the more demanding conditions, as part of the strategy to ensure a successful performance and to keep good accuracy levels, whereas Pre-HD participants tend to give more erroneous responses in the tasks with higher executive and memory load, and show a faster saccadic reaction time compared to controls. The changes in saccade timing (latency) observed in the clinical group might represent automatic processes and work as a proxy for the impulsivity and inhibitory control deficits often described in Huntington’s disease. This impulsivity-related response pattern matches the speed-accuracy tradeoff described by Heitz [84] where faster responses entail less accumulated evidence, and hence less informed decisions. This finding is also in line with the study of Vaportzis et al. [85] that reported that HD participants were affected differently than controls with respect to the competing goals of speed and accuracy. Moreover, these results seem to have similarities with the reflection impulsivity attributed to PD patients during rapid decision paradigms [86], that is, a tendency to “jump to conclusions” without gathering sufficient information [36].

Rao et al. [31] claim that response-inhibition failure in premanifest HD is associated with functional changes in inhibitory control, attentional reorienting, and motor-control systems. Because neural degeneration in HD begins in the basal ganglia, and saccadic suppression and inhibitory control mechanisms appear to be affected directly by these changes, measures of saccadic suppression, specifically, may be an effective early indicator of disease onset and impulsivity symptoms in premanifest HD, as response inhibition can serve as a “proxy” for the study of impulsivity and its neurobiological underpinnings [28, 44].

We hypothesized that an oculomotor paradigm with increment of executive and/or working-memory load might be more sensitive to the earliest HD-related changes if tapping onto the inhibition of saccades, since the frontostriatal circuitry is known to be affected one to two decades prior to estimated disease clinical onset [87–89]. This is relevant when searching for sensitive and low-cost markers of earliest functional changes due to HD neurodegenerative processes. In contrast to studies in healthy individuals [52, 54] we embedded the n-back memory component in the saccadic task, instead of a separate auditory or visual presentation of letters, respectively. We envisioned this would allow to discard interference from other sensory modalities and to better disentangle impairment in oculomotor inhibition in scope of HD neurodegeneration. Despite former findings of oculomotor alterations in premanifest HD [20, 34, 41–44, 46, 48–51, 53, 82], our study remains one of the few to have significant results on a sample of Pre-HD participants that are on average far from estimated clinical onset [20, 46, 50]. Furthermore, even though age is known to affect performance of reflexive and voluntary eye movements, both in healthy and clinical populations, previous studies have not controlled systematically for such effects, which might affect the positive results reported. Also, in former studies, the criterion for the categorization of premanifest and manifest HD individuals has been based on subjective confidence ratings (for example, see [49]), and not in a clear and standardized cut-off score as in the UHDRS-Motor scale [59].

At last, the application of pattern classification algorithms to oculomotor data has already shown promising results in differentiating premanifest HD individuals from control participants [90, 91], yet the interpretation of results in view of the dysfunction of inhibitory motor control remains elusive.

Regarding the conventional neuropsychological assessment results, the comparable cognitive baseline performance of the Pre-HD and Control participants in our study is in accordance with previous studies that did not detect differences between the cognitive profile of gene positive and gene negative/healthy control individuals [11, 17, 20, 51, 83, 92]. Even in large sample size studies (e.g., PREDICT-HD and TRACK-HD), the only robust cognitive deficits were detected in individuals that were close to estimated clinical onset (HD symptom presentation) and in the more executive demanding tests [19, 22, 45]. Our sample of Pre-HD participants was composed by individuals that were on average far from estimated clinical onset (73% had 15 or more years to the time of HD clinical diagnosis, according to Langbehn’s formula [58]), which might have had an important impact in our overall results (e.g. small effect sizes). Furthermore, these results suggest that the differences found in oculomotor performance between Pre-HD and CTRL individuals cannot be otherwise explained by the two groups having a distinct overt cognitive baseline.

Finally, the significant correlations found between specific oculomotor parameters and HD clinical and cognitive features reinforce the view that the saccadic behavior of Pre-HD individuals, particularly under more executively demanding conditions, reliably mirrors the often subtle and underestimated cognitive and motor alterations that characterize the premanifest stage of HD, and also gives important information about disease onset and progression. This is in line with former findings in premanifest HD individuals: impaired oculomotor functioning was shown to be associated to worse performance on cognitive tasks [24]; response accuracy in a visual processing task was found to be significantly correlated with an index of disease progression [23]; reaction time in a sequential button pressing task significantly associated with estimated time to disease onset [83]; increased error rates in antisaccade and memory guided saccade tasks were demonstrated to be associated to more abnormalities in the UHDRS motor scale and to a closer estimated disease onset [20, 49]; higher cognitive impairment was shown to be significantly related to increased oculomotor changes [93]; and, antisaccade error rate has been found to increase proportionally with disease progression [94]. Thus, quantitative measures of oculomotor inhibitory control and impulsivity such as the ones computed from the AS and MAS tasks of our study protocol seem to be sensitive indicators of the disease status and progression stage of premanifest HD individuals.

In conclusion, our results indicate that the temporal and spatial properties of oculomotor function in Pre-HD individuals reflect an imbalance between goal oriented and automatic behavior, due to early inhibitory control deficits. Moreover, our data suggest that the failure of the inhibitory control mechanisms that are involved in simple and complex oculomotor responses can induce an impulsive eye movement pattern in otherwise asymptomatic carriers of the genetic mutation that matches the HD executive dysfunction syndrome described by Rosenblatt [26]. Hence, saccadic timing and trajectory measures may be an effective early indicator of disease onset in HD, namely of motor disinhibition and impulsivity signs. Further, the manifestation of timing or spatial deviations in the saccadic behavior of premanifest HD individuals might depend on the task, and the levels of inhibition involved as well as executive load.

### Limitations

The small sample size makes it difficult to further subdivide the Pre-HD group into those far and close from estimated clinical onset. Large longitudinal studies like TRACK-HD or PREDICT-HD found the most significant differences between the cognitive performance of asymptomatic HD gene carriers and controls in those participants close to clinical onset [19, 22, 45]; this might indicate that a stratification is necessary if one wants to find robust evidence of cognitive changes in premanifest HD. Moreover, the relatively small sample size enrolled in our study prevents us from being able to generalize our results—further work is essential to validate and replicate our findings in a larger sample. Finally, the fact that significant differences were absent at the level of conventional neuropsychological test results between Pre-HD and CTRL participants leads us to hypothesize that the neuropsychological test battery used, even if extensive, was not sufficiently sensitive to the subtle and earliest changes that occur in HD cognition—subtle changes synonymous of small effect sizes, might need larger samples of premanifest gene carriers for testing novel hypotheses. Also, having a set of more ecological neuropsychological tests would probably help to better distinguish between the Pre-HD and CTRL groups, as cognitive assessment methods that resemble daily-life tasks have proven to be more successful at differentiating premanifest HD individuals far from estimated disease onset and controls [95, 96].

## Conclusion

Our saccadic task results suggest that the performance of Pre-HD individuals deteriorates when a fronto-executive or/and memory load is added to the task. Moreover, the Pre-HD group appears to have deficits in goal-oriented oculomotor behavior—more automatic responses or impulsivity at the cost of timed-strategy for accurate decision making. Our findings also suggest that specific horizontal saccadic parameters that enclose inhibition and memory demands seem to be accurate indicators of disease-related features in premanifest HD individuals. Hence, measures of inhibitory control mechanisms in the context of eye movement paradigms may provide sensitive markers of clinical disease onset in Huntington’s disease and help understand the neurobehavioral underpinnings of impulsivity as a trait of HD phenotype. Lastly, new quantitative tools that are able to detect the earliest disease-related changes and provide information about premanifest HD subtle signs and symptoms are thought to be extremely relevant for the design and implementation of interventional strategies aimed at delaying the onset or progression of Huntington’s disease.

## Supplementary information

**Additional file 1: Table S1.** Classes of medication for Premanifest HD (Pre-HD) and Control (CTRL) Groups.

**Additional file 2: Table S2.** Number of included and excluded participants after identification of valid trials per saccadic task (25% criterion).

**Additional file 3: Table S3.** Number of outliers per group for each of the four oculomotor parameters across the four saccadic tasks.

**Additional file 4: Table S4.** Significant differences in the Pre-HD group performance across the four saccadic tasks.

**Additional file 5: Table S5.** Correlations between the oculomotor results of the Pre-HD group.

## Data Availability

De-identified and analyzed data might be available upon reasonable request, and in accordance to informed consents and Ethics Committee, from the corresponding author.
