# Supplementary material for: The effect of impulsivity and inhibitory control deficits in the saccadic behavior of premanifest Huntington’s disease individuals
Source: Orphanet J Rare Dis. 2019 Nov 8;14:246. doi: 10.1186/s13023-019-1218-y (PMC6839196; doi:10.1186/s13023-019-1218-y)
Supplement: Supplementary file 1 — Additional file 1: Table S1. Classes of medication for Premanifest HD (Pre-HD) and Control (CTRL) Groups. [file 13023_2019_1218_MOESM1_ESM.doc]

**Additional file 1: Table S1 – Classes of medication for Premanifest HD (Pre-HD) and Control (CTRL) Groups**

| ***(number of participants on)*** | ***CTRL*** | ***Pre-HD*** |
| --- | --- | --- |
|  |  |  |
| No Medication | 22 | 11 |
| Antidepressants | 0 | 4 |
| Anxiolytics, Sedatives and Hypnotics | 0 | 2 |
| Antipsychotics | 0 | 0 |
| CTRL – Control participants; Pre-HD – Premanifest HD participants | | |
