# Supplementary material for: The effect of impulsivity and inhibitory control deficits in the saccadic behavior of premanifest Huntington’s disease individuals
Source: Orphanet J Rare Dis. 2019 Nov 8;14:246. doi: 10.1186/s13023-019-1218-y (PMC6839196; doi:10.1186/s13023-019-1218-y)
Supplement: Supplementary file 2 — Additional file 2: Table S2. Number of included and excluded participants after identification of valid trials per saccadic task (25% criterion). [file 13023_2019_1218_MOESM2_ESM.doc]

**Additional file 2: Table S2 – Number of included and excluded participants after identification of valid trials per saccadic task (25% criterion)**

| ***Task*** | ***Included*** | ***Excluded***  ***(less than 25% valid trials)*** | ***Excluded***  ***(did not perform the task)*** |
| --- | --- | --- | --- |
|  |  |  |  |
| **Prosaccade** | *22 CTRL* | *0 CTRL* | *0 CTRL* |
|  | 14 Pre-HD | 0 Pre-HD | 1 Pre-HD |
| **Antisaccade** | 22 *CTRL* | 0 *CTRL* | 0 *CTRL* |
|  | 14 Pre-HD | 0 Pre-HD | 1 Pre-HD |
| **Memory Prosaccade** | 21 *CTRL* | 1 *CTRL* | 0 *CTRL* |
|  | 14 Pre-HD | 0 Pre-HD | 1 Pre-HD |
| **Memory Antisaccade** | 20 *CTRL* | 2 *CTRL* | 0 *CTRL* |
|  | 13 Pre-HD | 1 Pre-HD | 1 Pre-HD |
|  |  |  |  |
| CTRL – Control participants; Pre-HD – Premanifest HD participants | | | |
