# Supplementary material for: The effect of impulsivity and inhibitory control deficits in the saccadic behavior of premanifest Huntington’s disease individuals
Source: Orphanet J Rare Dis. 2019 Nov 8;14:246. doi: 10.1186/s13023-019-1218-y (PMC6839196; doi:10.1186/s13023-019-1218-y)
Supplement: Supplementary file 3 — Additional file 3: Table S3. Number of outliers per group for each of the four oculomotor parameters across the four saccadic tasks. [file 13023_2019_1218_MOESM3_ESM.doc]

**Additional file 3: Table S3 – Number of outliers per group for each of the four oculomotor parameters across the four saccadic tasks**

|  | ***% Successful Trials*** | | ***% Direction Errors*** | | ***% Anticipatory Saccade Errors*** | | ***Latency*** | |
| --- | --- | --- | --- | --- | --- | --- | --- | --- |
|  | ***CTRL*** | ***Pre-HD*** | ***CTRL*** | ***Pre-HD*** | ***CTRL*** | ***Pre-HD*** | ***CTRL*** | ***Pre-HD*** |
| **PS** | 2 | 1 | 0 | 2 | 1 | 1 | 1 | 0 |
|  |  |  |  |  |  |  |  |  |
| **AS** | 1 | 0 | 2 | 0 | 0 | 2 | 1 | 0 |
|  |  |  |  |  |  |  |  |  |
| **MPS** | 1 | 1 | 1 | 1 | 2 | 0 | 1 | 0 |
|  |  |  |  |  |  |  |  |  |
| **MAS** | 0 | 0 | 2 | 0 | 3 | 0 | 0 | 1 |

CTRL – Control participants; Pre-HD – Premanifest HD participants

PS – Prosaccade; AS – Antisaccade; MPS – 1- or 2-back memory Prosaccade; MAS – 1- or 2-back memory Antisaccade

Successful trials – trials free of errors (%); Direction errors – resulting from a reflexive saccade in the opposite direction of the correct hit (%); Anticipatory saccade errors – resulting from a premature saccade: participant takes less than 80 ms to start the saccade (%); Latency – saccadic reaction time: time from stimulus appearance to the onset of the first saccade (milliseconds)
