# Supplementary material for: The effect of impulsivity and inhibitory control deficits in the saccadic behavior of premanifest Huntington’s disease individuals
Source: Orphanet J Rare Dis. 2019 Nov 8;14:246. doi: 10.1186/s13023-019-1218-y (PMC6839196; doi:10.1186/s13023-019-1218-y)
Supplement: Supplementary file 4 — Additional file 4: Table S4. Significant differences in the Pre-HD group performance across the four saccadic tasks. [file 13023_2019_1218_MOESM4_ESM.doc]

**Additional file 4: Table S4 –** **Significant differences in the Pre-HD group performance across the four saccadic tasks**

| ***Pre-HD*** | ***Successful Trials***  ***(n= 12)*** | | ***Direction Errors***  ***(n= 11)*** | | ***Anticipatory Saccade Errors***  ***(n= 11)*** | | ***Latency***  ***(n= 12)*** | |
| --- | --- | --- | --- | --- | --- | --- | --- | --- |
|  |  |  |  |  |  |  |  |  |
|  | ***Z*** | ***p*** | ***Z*** | ***p*** | ***Z*** | ***p*** | ***Z*** | ***p*** |
|  |  |  |  |  |  |  |  |  |
| **PS - AS** | -3.059 | 0.002* | -2.845 | 0.004* | -1.272 | 0.203 | -3.059 | 0.002* |
| **PS - MPS** | -1.334 | 0.182 | -2.851 | 0.004* | -2.371 | 0.018* | -3.059 | 0.002* |
| **PS - MAS** | -3.059 | 0.002* | -2.934 | 0.003* | -2.371 | 0.018* | -3.059 | 0.002* |
| **AS - MPS** | -2.845 | 0.004* | -2.667 | 0.008* | -1.761 | 0.078 | -3.059 | 0.002* |
| **AS - MAS** | -1.647 | 0.099 | -1.156 | 0.248 | -1.490 | 0.136 | -3.059 | 0.002* |
| **MPS - MAS** | -3.059 | 0.002* | -2.803 | 0.005* | -0.730 | 0.465 | -2.275 | 0.023* |

Pre-HD – Premanifest HD participants

PS – Prosaccade; AS – Antisaccade; MPS – 1- or 2-back memory Prosaccade; MAS – 1- or 2-back memory Antisaccade

Successful trials – trials free of errors (%); Direction errors – resulting from a reflexive saccade in the opposite direction of the correct hit (%); Anticipatory saccade errors – resulting from a premature saccade: participant takes less than 80 ms to start the saccade (%); Latency – saccadic reaction time: time from stimulus appearance to the onset of the first saccade (milliseconds)

Wilcoxon Signed Ranks Test * Significant differences p≤ 0.05
