# Supplementary material for: The effect of impulsivity and inhibitory control deficits in the saccadic behavior of premanifest Huntington’s disease individuals
Source: Orphanet J Rare Dis. 2019 Nov 8;14:246. doi: 10.1186/s13023-019-1218-y (PMC6839196; doi:10.1186/s13023-019-1218-y)
Supplement: Supplementary file 5 — Additional file 5: Table S5. Correlations between the oculomotor results of the Pre-HD group. [file 13023_2019_1218_MOESM5_ESM.doc]

**Additional file 5: Table S5 – Correlations between the oculomotor results in the Pre-HD group**

| ***Pre-HD*** | ***AS***  ***Successful Trials***  ***n = 14*** | | ***AS***  ***Direction Errors***  ***n = 14*** | | ***MAS***  ***Direction Errors***  ***n = 13*** | | ***MAS***  ***Latency***  ***n = 12*** | |
| --- | --- | --- | --- | --- | --- | --- | --- | --- |
|  | ***rho*** | ***p*** | ***rho*** | ***P*** | ***rho*** | ***p*** | ***rho*** | ***p*** |
|  |  |  |  |  |  |  |  |  |
| **PS** |  |  |  |  |  |  |  |  |
| **Successful Trials** | 0.771 | 0.001** | -0.676 | 0.008* | -0.138 | 0.654 | -0.182 | 0.572 |
| **Direction Errors** | -0.272 | 0.347 | 0.149 | 0.611 | 0.275 | 0.363 | -0.233 | 0.466 |
| **Anticipatory Errors** | -0.477 | 0.084 | 0.419 | 0.136 | 0.327 | 0.275 | -0.086 | 0.791 |
| **Latency** | -0.046 | 0.875 | -0.207 | 0.478 | -0.071 | 0.817 | 0.147 | 0.649 |
|  |  |  |  |  |  |  |  |  |
| **AS** |  |  |  |  |  |  |  |  |
| **Successful Trials** | 1.000 |  | -0.887 | 0.000** | -0.364 | 0.222 | -0.263 | 0.409 |
| **Direction Errors** | -0.887 | 0.000** | 1.000 |  | 0.383 | 0.197 | 0.347 | 0.269 |
| **Anticipatory Errors** | -0.837 | 0.000** | 0.747 | 0.002** | 0.356 | 0.233 | -0.105 | 0.746 |
| **Latency** | -0.460 | 0.098 | 0.260 | 0.370 | 0.093 | 0.762 | 0.441 | 0.152 |
|  |  |  |  |  |  |  |  |  |
| **MPS** |  |  |  |  |  |  |  |  |
| **Successful Trials** | 0.549 | 0.042* | -0.258 | 0.373 | -0.295 | 0.328 | 0.319 | 0.312 |
| **Direction Errors** | -0.251 | 0.386 | 0.288 | 0.318 | 0.271 | 0.370 | -0.152 | 0.638 |
| **Anticipatory Errors** | -0.539 | 0.047* | 0.284 | 0.325 | 0.513 | 0.073 | 0.431 | 0.161 |
| **Latency** | -0.282 | 0.329 | 0.176 | 0.547 | 0.049 | 0.873 | 0.629 | 0.028* |
|  |  |  |  |  |  |  |  |  |
| **MAS** |  |  |  |  |  |  |  |  |
| **Successful Trials** | 0.449 | 0.107 | -0.095 | 0.747 | -0.401 | 0.174 | 0.049 | 0.880 |
| **Direction Errors** | -0.385 | 0.173 | 0.264 | 0.361 | 1.000 |  | -0.196 | 0.542 |
| **Anticipatory Errors** | -0.262 | 0.366 | 0.054 | 0.855 | 0.322 | 0.283 | -0.303 | 0.339 |
| **Latency** | -0.154 | 0.599 | 0.077 | 0.793 | -0.115 | 0.707 | 1.000 |  |

PS – Prosaccade; AS – Antisaccade; MPS – 1- or 2-back memory Prosaccade; MAS – 1- or 2-back memory Antisaccade

Successful trials – trials free of errors (%); Direction errors – resulting from a reflexive saccade in the opposite direction of the correct hit (%); Anticipatory saccade errors – resulting from a premature saccade: participant takes less than 80 ms to start the saccade (%); Latency – saccadic reaction time: time from stimulus appearance to the onset of the first saccade (milliseconds)

* Correlation is significant at 0.05 level (two-tailed)

** Correlation is significant after Benjamini-Hochberg correction: AS % valid trails p≤ 0.001; AS % direction errors p≤ 0.002; MAS % direction errors p≤ 0.01; MAS latency p≤ 0.02)
